# A Rare Infratemporal Fossa Abscess of the Lateral Pterygoid

**DOI:** 10.7759/cureus.25391

**Published:** 2022-05-27

**Authors:** Benjamin S Daines, Rahul Varman, Joehassin Cordero

**Affiliations:** 1 Department of Otolaryngology - Head and Neck Surgery, Texas Tech University Health Sciences Center, Lubbock, USA

**Keywords:** infratemporal fossa, dental infection, facial abscess, lateral pterygoid, infratemporal abscess

## Abstract

The infratemporal fossa (ITF) contains many critical neurovascular structures and the muscles of mastication. Infections of the ITF are rare and carry significant pathologic implications due to nearby structures. Abscesses of the ITF often occur due to odontogenic spread and present with trismus and facial pain. We present the case of a 26-year-old male with an uncommon ITF abscess of the lateral pterygoid following maxillary wisdom tooth extraction. The abscess resolved following bedside and operative intraoral drainage in addition to intravenous antibiotic therapy. We highlight the rarity of lateral pterygoid abscesses and the importance of distinguishing ITF abscesses from other similarly presenting conditions such as temporomandibular joint dysfunction.

## Introduction

The infratemporal fossa (ITF) is a complex potential space harboring many critical structures. The space is bordered superiorly by the greater wing of the sphenoid bone, medially by the lateral pterygoid plate, laterally by the ramus of the mandible, anteriorly by the posterolateral maxillary sinus, and posteriorly by the tympanic part of the temporal bone [1]. The ITF contains multiple important structures including the maxillary artery, the pterygoid venous plexus, the mandibular nerve, the chorda tympani, and the muscles of mastication [1-3]. The major muscles of mastication in the ITF include the lateral pterygoid, which courses through the superior ITF, and the medial pterygoid found in the inferior ITF [1-3]. Due to these critical structures, the pathology of the ITF can result in trismus, dysgeusia, facial paresthesia, and difficulties with mastication [3].

Infection of the ITF with abscess formation is uncommon and often presents insidiously [4,5]. Infection most often arises from the contiguous spread of odontogenic infections via the masticator space to the ITF [4,5]. ITF abscesses can mimic other inflammatory disorders of the same anatomic region, such as temporomandibular joint (TMJ) dysfunction or temporal arteritis, leading to misdiagnosis [6-8]. Misdiagnosis is especially common with the abscess involvement of the muscles of mastication, leading to significant trismus and jaw pain [4,7]. Reports of such abscesses are rare, with only a single published case detailing a lateral pterygoid abscess [9]. To expand on the literature, here, we report the case of a 26-year-old male with an ITF abscess of the lateral pterygoid.

## Case presentation

A 26-year-old male with an insignificant medical history presented with left facial swelling. The swelling began two weeks ago after a bilateral maxillary wisdom tooth extraction. The swelling was accompanied by constant and dull pain localized to the left face with radiation to the left temple. The pain was exacerbated by jaw opening and was unrelieved by topical 2% oral lidocaine gel and oral ibuprofen. He endorsed trismus and low-grade fever but denied dyspnea and dysphagia. His symptoms persisted despite one week of oral amoxicillin (500 mg three times per day) followed by one week of oral clindamycin (300 mg three times per day). He denied any recent facial trauma or a history of oral infections. He endorsed daily recreational marijuana use and social drinking once a month but denied using tobacco products.

**Figure 1:**
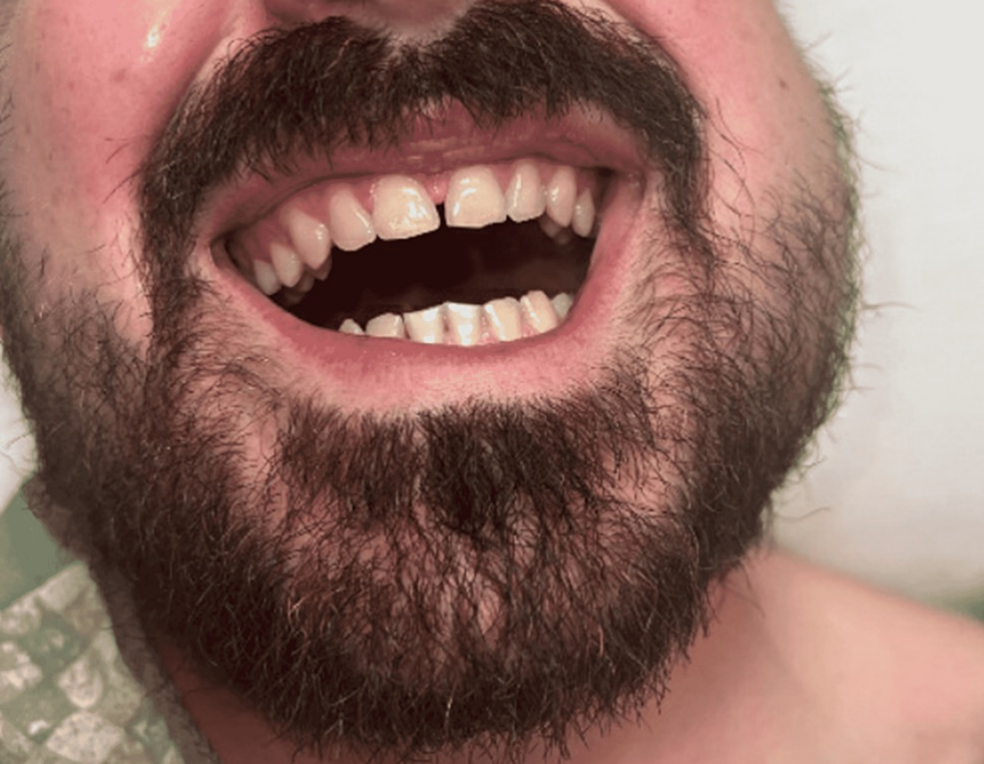
Left facial swelling with demonstrated trismus

**Figure 2:**
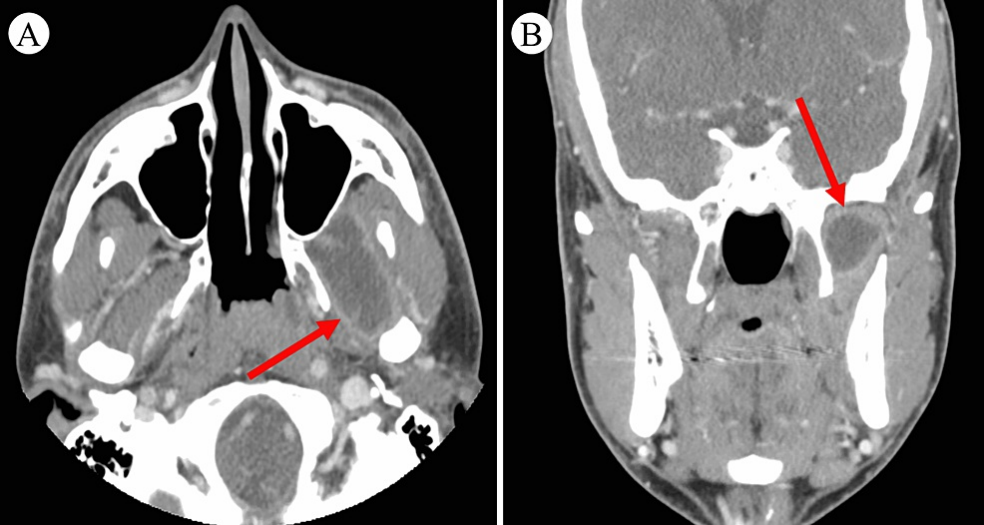
Maxillofacial CT demonstrating infratemporal fossa abscess of the lateral pterygoid (A) Axial CT of the abscess. (B) Coronal CT of the abscess.

On physical examination, the patient had significant swelling of the left preauricular area (Figure 1). The swelling was warm, fluctuant, and tender to palpation. The left buccal mucosa was tender without intraoral drainage or ulceration. The sites of wisdom tooth extraction were non-erythematous and without drainage bilaterally. Cranial nerves II through XII were intact with normal sensation over the entire face. The patient had no signs of dyspnea, dysphonia, or dysphagia. No cervical, submandibular, submental, or posterior occipital lymph nodes were palpable. The patient was febrile, and laboratory results demonstrated leukocytosis with a white blood cell (WBC) count of 17.3 K/µL. Maxillofacial CT scan demonstrated a 2.9 cm × 3.6 cm × 2.2 cm rim-enhancing fluid collection of the lateral pterygoid, indicative of an infratemporal fossa abscess (Figure 2). The abscess was drained at the bedside via intraoral approach due to the proximity to the buccal mucosa and to provide immediate pain relief, and 3 mL of purulent fluid was aspirated and sent for culture. A moderate reduction in facial swelling was appreciated post-procedure. The patient was admitted for observation and given empiric intravenous piperacillin-tazobactam (4.5 g every eight hours).

Despite the bedside aspiration, the patient experienced new-onset chills over the next day with a continued fever, swelling, trismus, and pain. Laboratory results demonstrated an increase in WBC count to 26.3 K/µL; therefore, operative incision and drainage were performed. An intraoral approach with an incision of the buccal mucosa overlying the abscess yielded a significant amount of purulent fluid. The procedure was well-tolerated, and the patient experienced an improvement in trismus, pain, and swelling over the next few days. His WBC count trended down to 9.85 K/µL during this time. Cultures of the abscess from the bedside and operative drainages both grew *Streptococcus anginosus* with resistance to clindamycin. Due to significant clinical and laboratory improvement, the patient was discharged with two weeks of oral amoxicillin-clavulanate (875-125 mg twice a day) and scheduled for otolaryngology clinic follow-up in two weeks.

## Discussion

Abscess of the ITF is a rare infectious process that most often occurs due to contiguous odontogenic spread [4]. Previous oral infections or tooth extractions are common with delays between weeks and months until ITF abscess formation [4,7,9]. The patient in this case had maxillary wisdom tooth removal two weeks before presenting with a significant ITF abscess. Oral infections of the maxilla tend to migrate superiorly into the temporal space where they may involve the lateral or medial pterygoid and form an abscess [10]. The spread of oral microbes such as *Streptococcus *sp. are commonly seen in other reports of ITF abscess like the patient presented in this case [2,11]. Often, no organisms are identified by abscess culture, which may be due to oral antibiotics taken prior to presentation [4,7,9]. Rarely, patients may present with atypical fungal or mycobacterial infections of the ITF [12].

Abscesses of the ITF most often present with trismus, facial pain, and difficulties with mastication due to direct irritation of the lateral and medial pterygoid [3]. The patient in this case presented with all of these symptoms, which have been shared by other patients in the reports of ITF abscesses [2,4,5]. Symptoms are often mistaken for TMJ dysfunction, leading to delays in diagnosis [6,7]. The involvement of the nearby structures including the mandibular nerve or chorda tympani may produce facial paresthesia, neuralgia, or dysgeusia [2,3]. Further spread may cause significant mortality due to cavernous sinus or deep cervical fascial involvement [4]. Early recognition with appropriate imaging via maxillofacial CT scan can accelerate treatment and prevent abscess expansion. Maxillofacial CT may also rule out pathology including tumors of the infratemporal fossa [13].

The lateral pterygoid is a rare abscess site in the ITF with only one other reported case [9]. This case shared many characteristics with the prior case, including trismus, fever, facial pain, antibiotic failure, and maxillary involvement [9]. The authors of the previous report opted for endoscopic transseptal drainage of the abscess via the maxillary sinus to prevent potential facial nerve damage or conductive hearing loss given the abscess location [9]. Other approaches have been utilized to drain abscesses of the ITF, including a transfacial approach [2,11,14] and a Caldwell-Luc approach [15]. Transfacial approaches to the ITF benefit from good exposure at the expense of facial excisions with potential cosmetic changes [16]. The Caldwell-Luc approach allows for greater instrumentation of the ITF with risks of facial swelling and nerve injury [17,18]. While operative drainage is the treatment of choice for ITF abscess, there is no current consensus in the literature on the preferred approach. The approach utilized may differ based on abscess location, size, and surgeon preference. Antibiotics are another mainstay of treatment, even in cases without culture growth [4,7]. The patient in this case successfully recovered with both operative drainage and intravenous antibiotics.

## Conclusions

The lateral pterygoid is a rare site of involvement for ITF abscesses. Inflammation of the lateral pterygoid due to abscess may cause significant trismus, facial pain, and difficulties with mastication. Lateral pterygoid abscesses can present similarly to TMJ dysfunction and must be differentiated early to prevent abscess expansion into nearby structures. Surgical drainage is the treatment of choice, and no consensus exists on the optimal approach. Future studies should investigate the optimal surgical approach and antibiotic therapy for ITF abscesses.
